# Complete blood count parameters as potential predictive factors of brain metastases in lung cancer

**DOI:** 10.3389/fonc.2025.1582788

**Published:** 2025-05-26

**Authors:** Karol Marschollek, Monika Kosacka, Anna Pokryszko-Dragan, Anna Brzecka-Bonnaud

**Affiliations:** ^1^ Department of Neurology, Wroclaw Medical University, Wrocław, Poland; ^2^ Department of Pulmonology and Lung Oncology, Wroclaw Medical University, Wrocław, Poland

**Keywords:** lung cancer, NSCLC, complete blood count, NLR, brain metastases

## Abstract

**Introduction:**

Brain metastases are common and devastating complication of the lung cancer (LC) but predictive biomarkers for their risk are still lacking.

**Objectives:**

To analyze the relationships between complete blood count (CBC)-based and selected biochemical indices and occurrence of brain metastases in patients diagnosed with LC.

**Patients and methods:**

The study was based on retrospective analysis of medical records of 217 patients diagnosed with LC and undergoing follow-up in one specialist center. Clinical and laboratory data on admission were determined, including: neutrophil-to-lymphocyte ratio (NLR), platelet-to-lymphocyte ratio (PLR), lymphocyte-to-monocyte ratio (LMR), neutrophil-to-platelet ratio (NPR) and red cell distribution width-to-platelet ratio (RPR) and selected biochemical parameters. Relationships were evaluated between these parameters and occurrence of brain metastases, other distant metastases and death within 12 months of follow-up.

**Results:**

168 patients had the follow-up data for 6 months, and 128 - for 12 months. Brain metastases were detected in 41 patients and 1-year mortality rate was 17.61%. Patients who developed brain metastases during 12 months had significantly higher baseline NLR (4.66 vs 2.75, p<0.001), PLR (170.83 vs 142.42, p=0.03) and lower LMR (1.61 vs 2.33, p=0.008).In univariate analysis, higher leukocyte count (HR 1.08, p=0.016), neutrophil count (HR 1.11, p=0.0036), NLR (HR 1.09, p=0.005), d-dimer levels (HR 1.0002, p=0.0043), and lower LMR (HR 0.67, p=0.018) were significantly associated with the risk of developing brain metastases. Liver metastases were associated with lower LMR (1.69 vs 2.29, p=0.04), while metastases to the other lung – with lower PLR (126.52 vs 161.8, p=0.02) and higher LMR (2.51 vs 1.96, p=0.02) and RPR (0.184 vs 0.154, p=0.03). No significant relationships were found between CBC-based indices and mortality.

**Conclusions:**

CBC-based indices could be useful and easily accessible predictive markers of brain metastases in the patients with lung cancer.

## 1 Introduction

Lung cancer is the most common cause of metastases to the central nervous system (CNS) and accounts for about 39-56% of all brain metastases (1). Due to raised intracranial pressure and focal symptoms of neurological deficit, metastatic brain tumors are associated with devastating impact on the patient’s condition as well as with poor prognosis. The overall median survival time since the time of diagnosis of brain metastases was estimated at 15.2 months for adenocarcinoma and 9.2 months for other types of non-small-cell lung cancer (NSCLC) (2). Thus, early identification of risk factors for neoplastic dissemination to CNS seems crucial for monitoring of the patients and optimal therapeutic decisions, aimed at the improvement of prognosis.

The inflammatory process is one of the relevant mechanisms of tumor progression and dissemination (3), therefore markers of the inflammatory response have been intensively studied for their predictive value in lung cancer. Particular attention has been paid to indices based on complete blood count (CBC), especially because of their accessibility and ease of identification. The best-known indicator with proven usefulness is the NLR (neutrophil-to-lymphocyte ratio), considered a prognostic factor in patients with solid tumors (4). In NSCLC, NLR was shown to correlate negatively with mean survival time (5), correspond with the prognosis after curative surgical resection (6) and response to chemotherapy (7) or immunotherapy (8). Other potentially prognostic markers that have been assessed in patients with lung cancer include PLR (platelet-to-lymphocyte ratio) (9) and LMR (lymphocyte-to-monocyte ratio) (10). Prognostic value in several malignancies was also suggested for other CBC indices such as red cell distribution width-to-platelet ratio (RPR) (11, 12) or neutrophil-to-platelet ratio (NPR), also considered indicators of an acute inflammatory response (13). These indices can be applied as single markers or as components of diagnostic/prognostic panels, e.g. advanced lung cancer inflammation index (ALI), which includes: body mass index, serum concentration of albumin and NLR, and has been used as a predictor of response to treatment with PD-L1 inhibitors (14). However, there is little evidence for the predictive value of these indices with regard to the occurrence of brain metastases in lung cancer (15, 16), as they have not been consistently investigated in this field.

## 2 Aim

The aim of the study was to analyze the relationships between CBC – based and selected biochemical indices and the occurrence of brain metastases in patients diagnosed with lung cancer and to evaluate predictive usefulness of these indices.

## 3 Patients and methods

### 3.1 Patients

The study was based on the retrospective analysis of the medical records from the Department of Pulmunology and Lung Oncology of the Lower Silesian Centre of Oncology, Lung Diseases and Haematology (Wroclaw, Poland). Patients diagnosed with lung cancer between 2016 and 2022, with documented follow-up for at least 3 months (unless they died before reaching this time point), were included in the study group. Exclusion criteria comprised active infection, concomitant inflammatory/autoimmune disorders, other malignancies on admission and current corticosteroid use, as well as lack of brain imaging results (computed tomography - CT or magnetic resonance imaging - MRI) in the documented follow-up. The final cohort consisted of 217 patients, aged 41–91 years. The diagnosis of lung cancer in all patients was confirmed with histopathological examination.

Age, sex, clinical and laboratory findings on admission were extracted from each patient’s medical records and anonymized/coded for further analysis. The following laboratory findings were determined: CBC with automated differential counts, serum level of C-reactive protein (CRP), d-dimers, glucose, electrolytes, parameters of kidney and liver function. On the basis of CBC, the following indices were calculated: NLR, PLR, LMR, NPR and RPR. Clinical data included histopathological subtype of lung cancer (small-cell lung cancer – SCLC, and NSCLC: adenocarcinoma, squamous cell carcinoma, large cell carcinoma, undifferentiated), TNM Classification of Malignant Tumours staging at the time of diagnosis, and comorbidities (**Table 1**). Relationships were evaluated between CBC-based indices (and other initial laboratory findings) and occurrence of brain metastases within 6 and 12 months of follow-up. Other outcomes of the lung cancer included in the analysis were metastases to other organs and death.

**Table 1:** Characteristics of the study group.

Characteristics	No (%) of patients
Gender	
Female	92 ( 42.4%)
Male	125 ( 47.6%)
Histopathological subtype of lung cancer	
NSCLC:	184 (84.8%)
Adenocarcinoma	71 (32.7%)
Squamous cell carcinoma	80 (36.9%)
Large cell carcinoma	2 (0.9%)
Undifferentiated	31 (14.3%)
SCLC	33 (15.2%)
TNM staging	
T1	13 (5.99%)
T2	35 (16.13%)
T3	34 (15.67%)
T4	121 (55.76%)
N0	27 (12.44%)
N1	32 (14.75%)
N2	91 (41.94%)
N3	51 (23.50%)
M0	117 (53.92%)
M1:	84 (38.71%):
Distant metastases location	
CNS	22
spine/bones	13
other lung	21
liver	16
adrenal glands	8
skin	2
breast	1
pancreas	1
Type of treatment	
Radiotherapy	119 (54.8%)
Chemotherapy	158 (72.8%)
Surgery	40 (18.4%)
Immunotherapy	36 (16.6%)
Comorbidities	
Arterial hypertension	120 (55.3%)
COPD	58 (26.7%)
Diabetes	43 (19.8%)
Ischemic heart disease	31 (14.3%)
Atrial fibrillation	21 (9.7%)
Heart failure	10 (4.6%)
Hypothyroidism	24 (11.1%)
History of ischemic stroke	11 (5.1%)
Epilepsy	5 (2.3%)
Chronic Kidney Disease	10 (4.6%)
Liver cirrhosis	1 (0.5%)

NSCLC, Non-small cell lung cancer; SCLC, Small cell lung cancer; COPD, Chronic obstructive pulmonary disease.

All the procedures were performed in accordance with the Declaration of Helsinki and its further amendments. Informed consent was not required for this study as it was conducted retrospectively and involved the analysis of previously collected data. The project of the study was approved by the Wroclaw Medical University Bioethics Committee (approval no. KB-918/2021).

### 3.2 Statistical analysis

Categorical variables were presented as numerical and percentage values and continuous ones – as the mean and standard deviation (SD) or median and interquartile range. The χ2 test, Student’s t-test and Pearson’s correlation coefficient were used for normally distributed data, and non-parametric tests (Mann–Whitney U test and Spearman’s correlation coefficient) were used for other variables.

After establishing differences between analyzed groups, Cox proportional hazards regression was used to evaluate the association between potentially significant laboratory variables and occurrence of brain metastases during one year of follow up. Time-to-event was measured in months, defined as the duration from the enrollment to the event of interest (death or the diagnosis of metastases) or censoring. All variables were assessed in univariable Cox models. Hazard ratios (HRs), 95% confidence intervals (CIs), and corresponding p-values were reported for each predictor. Model assumptions, including the proportional hazards assumption, were assessed using Schoenfeld residuals. The linearity assumption for continuous variables was evaluated through Martingale residual plots.

To determine suggested threshold values for continuous predictors identified as significant in the Cox regression, receiver operating characteristic (ROC) curve analysis was performed. The area under the curve (AUC) was reported, and cut-off values for high risk of developing brain metastases were selected based on the Youden index (calculated as: specificity+sensitivity – 1).The p-values <0.05 were considered statistically significant. The analysis was performed with STATISTICA v. 13.0 software (StatSoft Polska, Cracow, Poland).

## 4 Results

### 4.1 Clinical outcomes

**Figure 1:**
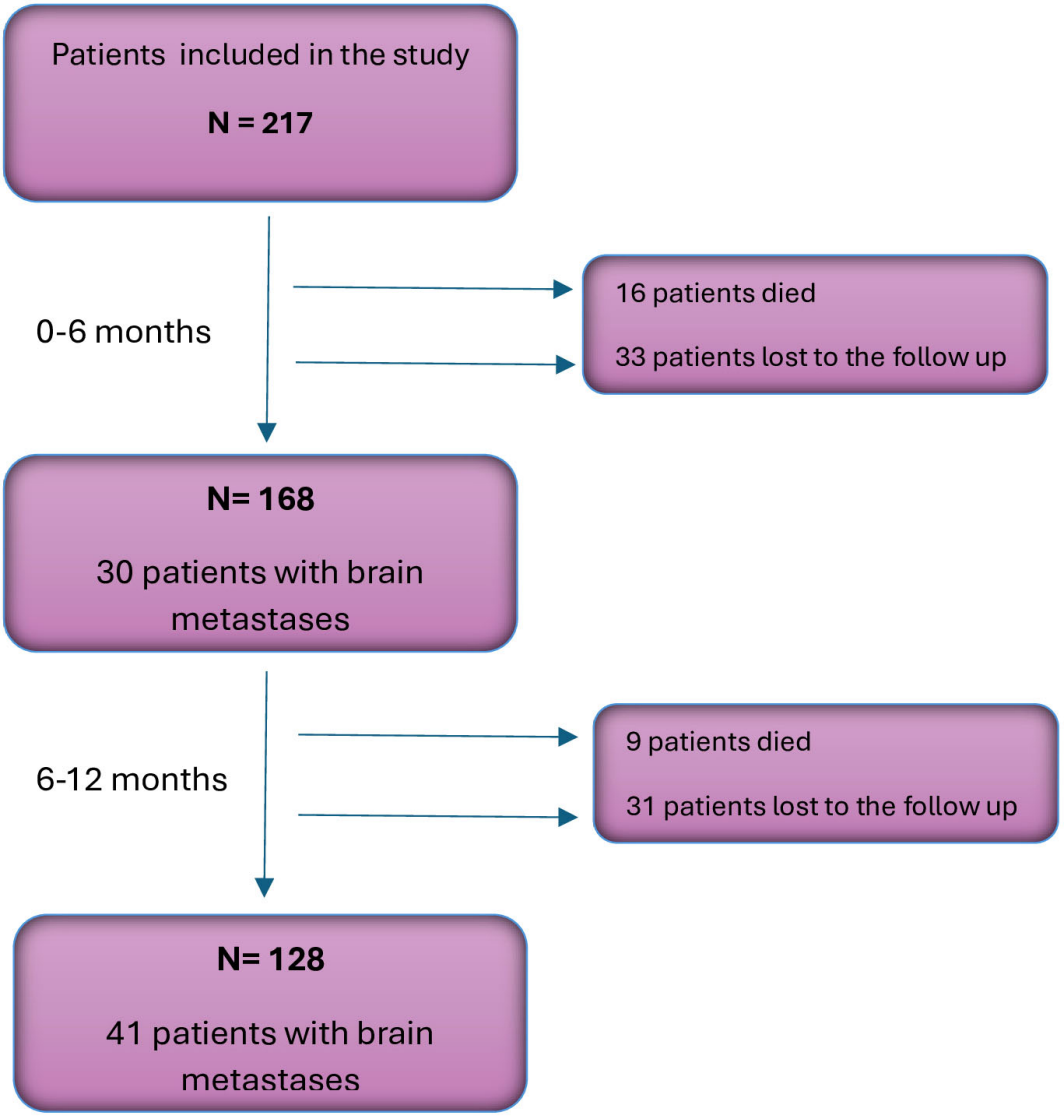
Flowchart illustrating changes in the structure of the study group during the follow-up.

Of the 217 patients included in the study, 168 had the follow-up data for 6 months, and 128 patients - for 12 months. During the analyzed follow-up period, CNS metastases were detected in 41 patients (32.03%). Other sites of metastases included: spine/bones (n=26, 20.31%), the other lung (n=23, 17.97%), liver (n=19, 14.84%), adrenal glands (n=11, 8.59%), skin (n=3, 1.38%), breast (n=1, 0.46%) and pancreas (n=1, 0.46%). **Figure 1** shows changes in the structure of the study group during the follow-up.

### 4.2 Baseline laboratory findings and occurrence of brain metastases

Values of the baseline laboratory tests were compared between the patients in whom brain metastases were or were not detected during the follow-up period. The results of the comparison with regard to the time of the metastasis occurrence are presented in **Table 2**. At baseline, patients who developed brain metastases during subsequent 12 months, had significantly higher median total leukocyte and neutrophil counts, red cell distribution width (RDW) and lower median eosinophil count, as well as higher alanine aminotransferase (ALT), d-dimer, CRP and lactate dehydrogenase (LDH) serum levels. Although patients with brain metastases revealed during first 6 months had significantly lower lymphocyte counts and mean platelet volume (MPV) at baseline, this difference did not reach statistical significance with regard to the assessment after 12 months. Analysis of CBC-based indices revealed significantly higher values of NLR, PLR and lower LMR in those patients who developed brain metastases at any time point during the follow-up.

**Table 2:** Baseline blood- based indices regarding a subsequent occurrence of brain metastases during the follow-up.

Parameter	Brain metastases within 6 months			Brain metastases within 12 months		
*Median (interquartile range)*	Yes (n=30)	No (n=138)	p	Yes (n=41)	No (n=87)	p
Total Leukocyte count, n × 10^9^/L	10.90 (7.88-13.88)	8.40 (7.30-10.28)	0.005	10.50 (7.90-11.60)	8.20 (7.15-10.35)	0.008
Neutrophil count, n × 10^9^/L	7.55 (5.92-10.20)	5.54 (4.58-7.16)	<0.001	6.81 (5.63-8.42)	5.46 (4.39-7.12)	<0.001
Lymphocyte count, n × 10^9^/L	1.25 (0.96-2.10)	1.86 (1.53-2.30)	0.004	1.54 (1.05-2.14)	1.90 (1.56-2.30)	0.05
Eosinophil count, n × 10^9^/L	0.05 (0.01-0.16)	0.14 (0.06-0.21)	0.004	0.08 (0.02-0.20)	0.14 (0.07-0.20)	0.049
RDW SD, fl	46.00 (43.30-50.08)	44.05 (41.90-46.65)	0.015	45.00 (42.90-48.80)	43.90 (41.90-46.45)	0.049
NLR	6.30 (3.83-7.89)	2.92 (2.18-4.41)	<0.001	4.66 (3.05-7.13)	2.75 (2.17-4.31)	<0.001
PLR	201.90 (148.26-293.46)	152.02 (110.28-204.13)	0.001	170.83 (142.99-237.98)	142.42 (107.87-206.40)	0.03
LMR	1.51 (1.21-2.38)	2.36 (1.68-2.97)	<0.001	1.61 (1.39-2.51)	2.33 (1.65-2.99)	0.008
NPR	0.03 (0.02-0.04)	0.02 (0.02-0.03)	0.002	0.02 (0.02-0.03)	0.02 (0.02-0.02)	0.01
RPR	0.16 (0.13-0.24)	0.16 (0.13-0.19)	0.81	0.16 (0.13-0.20)	0.16 (0.13-0.20)	0.91
	Yes (n=30)	No (n=137)	p	Yes (n=41)	No (n=86)	p
Creatinine, mg/dL	0.68 (0.56-0.84)	0.80 (0.68-0.96)	0.02	0.74 (0.64-0.92)	0.81 (0.67-0.93)	0.21
Sodium, mmol/L	137 (136-139)	140 (137-142)	0.004	138 (136-142)	139.5 (137-141)	0.39
Potassium, mmol/L	4.42 (4.14-4.81)	4.44 (4.14-4.73)	0.72	4.41 (4.14-4.66)	4.44 (4.10-4.65)	0.83
	Yes (n=19)	No (n=61)	p	Yes (n=23)	No (n=36)	p
Urea, mg/dL	33.8 (24.45-44.55)	33.3 (24.6-40.8)	0.48	32.9 (24.45-41.40)	32.10 (24.12-37.35)	0.34
	Yes (n=28)	No (n=124)	p	Yes (n=38)	No (n=77)	p
activated partial thromboplastin time aPTT; s	25.11 (23.30-26.76)	26.55 (24.95-29.12)	0.016	25.81 (23.84-27.08)	26.32 (25-29.12)	0.05
	Yes (n=27)	No (n=79)	p	Yes (n=32)	No (n=49)	p
d-dimer, ng/mL	1081.7 (596.80-2606.6)	633.9 (414.10-1126.40)	0.015	877.40 (531.50-2400.60)	622.0 (411.60-974.70)	0.02
	Yes (n=29)	No (n=128)	p	Yes (n=38)	No (n=80)	p
alanine aminotransferase (ALT), U/L	22 (17-37)	15 (11-21.25)	<0.001	19.5 (13.5-34.75)	15 (11-20.25)	0.01
	Yes (n=29)	No (n=130)	p	Yes (n=38)	No (n=81)	p
aspartate aminotransferase (AST), U/L	19 (16-25)	17 (14-21.75)	0.25	19 (15.25-24.75)	17 (14-21)	0.25
	Yes (n=12)	No (n=28)	p	Yes (n=14)	No (n=15)	p
Lactate dehydrogenase (LDH), U/L	275.6 (181.2-324.7)	175.3 (151-213.9)	0.06	239.1 (164.7-318.2)	167.6 (142.5-184.2)	0.015
	Yes (n=28)	No (n=104)	p	Yes (n=34)	No (n=64)	p
C reactive protein (CRP), mg/L	13.22 (7.36-37.49)	7.65 (3.46-20.5)	0.09	11.16 (5.35-31.33)	5.63 (2.83-18.09)	0.04

Variables for which significant differences were detected at baseline were included in further analysis as potential predictors of brain metastases.

The univariate analysis revealed significant associations between the occurrence of brain metastases and total leukocyte count (HR 1.08, 95% CI 1.02-1.15, p=0.016), neutrophil count (HR 1.11, 95% CI 1.03-1.19, p=0.0036), NLR (HR 1.09, 95% CI 1.03-1.19, p=0.005), LMR (HR 0.67, 95% CI 0.48-0.93, p=0.018) and d-dimer (HR 1.0002, 95% CI 1.0001-1.0004, p=0.0043) levels.

To determine the optimal thresholds for predicting higher risk of brain metastases, receiver operating characteristic (ROC) curve analysis was performed for aforementioned variables. The area under the ROC curve (AUC) was highest for NLR (0.70, 95% CI: 0.60–0.80), indicating good discriminatory ability. The optimal cut-off value for NLR, identified using the Youden Index, was 4.56, corresponding to a sensitivity of 54% and a specificity of 80% (Youden Index = 0.34).

The results of Cox proportional hazards regression and ROC/AUC analysis are summarized in **Table 3**.

**Table 3:** Results of univariable Cox regression analyses for CBC indices and other laboratory predictors of occurrence of brain metastases.

Variable	HR (95% CI)	p	Suggested threshold	Youden index	AUC (95% CI)
Total Leukocyte count	1.08 (1.02-1.15)	0.016	10.5	0.29	0.65 (0.54-0.75)
Neutrophil count	1.11 (1.03-1.19)	0.0036	5.56	0.34	0.68 (0.58-0.78)
Lymphocyte count	0.69 (0.44-1.09)	0.114			
Eosinophil count,	0.36 (0.04-3.21)	0.357			
RDW SD	1.04 (0.98-1.10)	0.212			
NLR	1.09 (1.03-1.19)	0.005	4.56	0.34	0.70 (0.60-0.80)
PLR	1.001 (0.999-1.003)	0.316			
LMR	0.67 (0.48-0.93)	0.018	1.66	0.31	0.65 (0.54-0.75)
d-dimer	1.0002 (1.0001-1.0004)	0.0043	1987	0.23	0.65 (0.53-0.77)
alanine aminotransferase (ALT)	1.005 (1.001-1.009)	0.0145*			
Lactate dehydrogenase (LDH)	0.999 (0.998-1.001)	0.769			
C reactive protein (CRP)	1.003 (0.998-1.009)	0.181			

Hazard ratios (HRs) with corresponding 95% confidence intervals (CIs) and p-values as well as suggested thresholds for high risk of brain metastases selected based on the Youden index are presented for each predictor.

*The model has not reached statistical significance.

### 4.3 Baseline laboratory findings and other localization of metastases

An analysis of baseline laboratory findings was also performed with regard to the occurrence of metastases to other organs within 12 months of the follow-up. Liver metastases were associated with higher baseline level of CRP and urea, and lower LMR.

Patients with metastases to the other lung had lower PLR and platelet count as well as higher LMR and RDW/platelet ratio. There were no significant differences in baseline laboratory findings between patients with or without bone metastases.

### 4.4 Mortality

Mortality rate in the study group during one year of follow-up was 17.61% (n=25). Patients who died during the follow-up, presented at baseline with significantly lower median eosinophil count and serum sodium level and higher median CRP level, while no statistically significant differences were found for CBC indices.

The baseline CBC parameters with regard to the occurrence of particular metastases and death during the follow-up are summarized in **Table 4**.

**Table 4:** Analysis of baseline CBC-based indices regarding a subsequent occurrence of other metastases and death during 12 months of follow-up.

Parameter	Liver metastases within 12 months			Bone metastases within 12 months			Lung metastases within 12 months			Death within 12 months		
*Median (interquartile range)*	Yes (n=19)	No (n=109)	p	Yes (n=26)	No (n=102)	p	Yes (n=23)	No (n=105)	p	Yes (n=25)	No (n=117)	p
Total Leukocyte count, n × 10^9^/L	9.7 (7.3-12.2)	8.4 (7.3-10.9)	0.54	9.45 (7.3-11.3)	8.4 (7.3-11.2)	0.71	8.1 (7-10.4)	8.6 (7.4-11.3)	0.24	10.05 (7.5-12.25)	8.4 (7.3-11.1)	0.29
Neutrophil count, n × 10^9^/L	6 (4.93-8.42)	5.59 (4.62-7.19)	0.23	5.65 (4.81-8)	5.79 (4.55-7.2)	0.58	5.47 (4.51-6.85)	5.89 (4.68-7.99)	0.21	6.42 (5.28-9.65)	5.73 (4.65-7.51)	0.14
Lymphocyte count, n × 10^9^/L	1.39 (1.05-2)	1.88 (1.48-2.26)	0.07	1.57 (1.24-2.27)	1.82 (1.39-2.24)	0.34	1.97 (1.59-2.5)	1.78 (1.23-2.22)	0.2	1.48 (1.17-2.26)	1.81 (1.39-2.22)	0.12
Eosinophil count, n × 10^9^/L	0.13 (0.03-0.19)	0.13 (0.06-0.20)	0.60	0.13 (0.06-0.22)	0.135 (0.06-0.2)	0.88	0.11 (0.06-0.19)	0.14 (0.06-0.2)	0.74	0.06 (0.03-0.15)	0.14 (0.07-0.2)	0.03
RDW SD, fl	42.9 (41.7-46.7)	44.4 (42.1-47.1)	0.49	44.2 (42.1-46.7)	44.2 (41.9-47.1)	0.97	45.9 (42-48.1)	44 (41.9-46.7)	0.33	45 (42.2-49.5)	44 (41.9-47)	0.22
NLR	4.38 (3.39-6.35)	3.05 (2.24-5.13)	0.06	4.14 (2.38-5.47)	3.09 (2.24-5.15)	0.18	2.67 (2.25-3.44)	3.41 (2.26-5.58)	0.13	4.34 (2.77-6.59)	3.12 (2.26-5.15)	0.06
PLR	200.43 (136.78-276.19)	150 (110.69-204.37)	0.10	163.31 (127.92-224.04)	151.02 (110.40-220)	0.60	126.52 (93.2-181.3)	161.8 (126.24-227.16)	0.02	176.07 (147.10-276.19)	153.66 (115.11-220)	0.12
LMR	1.69 (1.40-2.33)	2.29 (1.55-2.97)	0.04	1.75 (1.53-2.44)	2.29 (1.47-2.99)	0.17	2.51 (2.16-3.38)	1.96 (1.44-2.87)	0.02	2.35 (1.69-2.51)	2.15 (1.47-2.91)	0.91
NPR	0.022 (0.020-0.027)	0.021 (0.017-0.026)	0.30	0.022 (0.018-0.028)	0.021 (0.017-0.026)	0.38	0.022 (0.019-0.026)	0.021 (0.017-0.026)	0.51	0.019 (0.016-0.028)	0.021 (0.017-0.026)	0.91
RPR	0.173 (0.103-0.213)	0.157 (0.131-0.198)	0.79	0.167 (0.143-0.203)	0.158 (0.127-0.198)	0.54	0.184 (0.154-0.213)	0.154 (0.124-0.186)	0.03	0.155 (0.128-0.198)	0.157 (0.127-0.194)	0.91

## 5 Discussion

The aim of the current study was to identify parameters based on easily available peripheral blood tests with potential predictive value for brain metastases in patients with lung cancer. We found significant association between developing brain metastases within 6 and 12 months of follow-up and baseline leukocyte subpopulation counts and blood cell subsets ratios, as well as selected biochemical parameters.

Increased number of neutrophils and lowered number of lymphocytes in the patients with brain metastases seem consistent with the current views on the role of neutrophils in the progression of the neoplasm. Neutrophils - especially their population defined as tumor associated neutrophils (TANs) - take part in many processes related to the development of neoplasms, including systemic immunosuppression (by inhibiting T-cells activity and proliferation) and production of specific cytokines, chemokines and proteases, which modulate tumor microenvironment (TME), contribute to local angiogenesis and dissemination of neoplastic cells (17, 18). Interestingly, TANs can adopt either anti-tumor (N1) or pro-tumor (N2) phenotypes. This bilateral functionality depends on various factors within the TME, and pro-inflammatory factors, particularly tumor growth factor β (TGF- β), are considered to promote the latter (19). One of possible functions of TANs is the remodeling of the extracellular matrix through the secretion of proteases such as neutrophil elastase, matrix metalloproteinases and cathepsin G as well as promoting epithelial–mesenchymal transition via the release of reactive oxygen species (ROS) and cytokines such as IL-8, enhancing tumor cells invasiveness (20). Another possible mechanism enhancing metastatic activity is by aggregating with circulating tumor cells (CTCs) and protecting them from immune detection while in circulation. In TME, neutrophil extracellular traps (NETs) can trap CTCs and provide a vector for their adhesion to endothelial surfaces, promoting extravasation and colonization. NET formation has been associated with microvascular damage and enhanced metastatic burden in preclinical models (20). Moreover, TANs also contribute to the formation of the pre-metastatic niche by releasing pro-inflammatory mediators (such as IL-1β, TNF-α) and promoting vascular permeability, which primes target tissues for colonization (21).

This mechanism of action may be highly relevant in the context of brain metastases, where the integrity of the blood-brain barrier (BBB) is a critical limiting factor. In the setting of systemic inflammation and tumor progression, TANs and associated cytokines play a key role in increasing BBB permeability. Several studies have demonstrated that pro-inflammatory cytokines disrupt tight junction proteins such as occludin and claudin-5 in brain microvascular endothelial cells (22). This disruption leads to increased paracellular permeability, facilitating the migration of tumor cells across the BBB. Additionally, TAN-derived ROS and nitric oxide further compromise endothelial barrier function.

In turn, a lower number of lymphocytes, responsible for the specific immune response, has been found to correlate with a worse overall survival (OS) and progression free survival (PFS) in solid tumors, including lung cancer (23, 24). Another parameter associated with brain metastases in the study group was an increased baseline RDW, an indicator of the variability of red blood cell size. This may further strengthen the hypothesis of the inflammatory background of metastatic activity, as RDW was reported to correlate positively with established inflammatory markers (25). Furthermore, RDW was also considered as a negative prognostic marker in NSCLC (26). Song et al. found significantly higher values of RDW in patients with NSCLC compared with healthy individuals, as well as a positive correlation between RDW and TNM stage. Additionally, in the patients who developed brain metastases, we observed lower baseline eosinophil counts. This finding seems particularly interesting in view of other reports on lung cancer, which indicate a role of eosinophil level as a predictor of response to therapy with immune checkpoint inhibitors (27, 28).

Indices based on CBC have been extensively studied and encouraged for their use in the assessment of the intensity of systemic inflammatory processes. Their diagnostic and prognostic utility has been confirmed in various autoimmune diseases, such as systemic lupus erythematosus (29), Sjogren syndrome (30) or ulcerative colitis (31), but also in several types of cancer (32–35). The most popular among these indices, NLR, reflects a disproportion between leukocyte subpopulations. Increased NLR values have already been associated with a worse prognosis and poor response to treatment in lung cancer (5, 36).

Some authors reported correlation of other indices (mainly PLR and MLR) with survival in patients with lung cancer (9, 10) and in patients with metastatic brain tumors in general (37, 38). However, data on prognostic utility of these indices for CNS metastases from the lung cancer is scarce.

Indeed, we found that patients with occurrence of brain metastases within a year since lung cancer was diagnosed, had a significantly higher baseline NLR value than patients who did not develop metastases. Similar association was observed for lower baseline values of LMR and (with lower but still relevant level of significance) higher baseline PLR.

Limited evidence on the investigated relationships is available in the literature. Multivariate analysis performed by Koh et al. (16) in patients with stage IV NSCLC revealed that patients with high NLR at diagnosis had higher cumulative incidence of subsequent brain metastases, with a threshold value of NLR ≥4.95 as their significant independent predictor. In another study, that included patients with lung adenocarcinoma (15), elevated levels of NLR were independently associated with an increased risk of the presence of brain metastases on admission. Similar findings were reported for patients with lung adenocarcinoma treated with radical surgery, with higher baseline PLR value related to increased risk of brain metastases during the follow-up (median - 30 months) (39).

Apart from CBC-based indices, we also investigated basic biochemical parameters. Significantly increased baseline level of CRP and LDH in the patients with subsequent brain metastases seem consistent with putative inflammatory/immune-mediated background for dissemination of neoplasm. CRP is a model inflammatory protein, and lactate dehydrogenase has been recently viewed as a pivotal modulator of immunogenicity and metabolic activity of various neoplasms (40). A significantly higher baseline level of d-dimers in these patients is primarily associated with dysfunction of the coagulation system during cancer progression and has been also associated with a worse OS and PFS in patients with lung cancer (41). We have also noted a significantly higher median levels of alanine aminotransferase in patients with subsequent brain metastases, which might reflect early concomitant hepatic cells destruction. However, the difference in baseline levels of aspartate aminotransferase between the subgroups of patients did not reach the statistical significance.

In this exploratory analysis, we identified several parameters that showed significant associations with the risk of developing brain metastases, including total leukocyte count, neutrophil count, neutrophil-to-lymphocyte ratio (NLR), lymphocyte-to-monocyte ratio (LMR), and d-dimer levels. Among these markers, NLR demonstrated the highest discriminatory ability in ROC curve analysis (AUC = 0.70), with an optimal cut-off of 4.56 providing moderate sensitivity (54%) and relatively high specificity (80%).

However, while these results suggest a possible predictive utility of inflammatory markers - particularly NLR - in identifying patients at elevated risk of brain involvement, due to the retrospective and single-center design of this study, the overall discriminative performance may be not sufficient for clinical application at this stage. Therefore the cut-off thresholds should be interpreted cautiously. We believe the findings may be valuable as hypothesis-generating, and further validation in larger, prospective cohorts is necessary before drawing conclusions or recommending integration into clinical decision-making.

It seems interesting that unlike CNS involvement, metastases to the other organs in the study group did not show such relevant relationships with baseline CBC indices. For those with or without liver metastases, only the difference in baseline LMR values reached statistical significance (insignificant trend was noticeable for total lymphocyte count and NLR). Furthermore, lower baseline eosinophil count was the only parameter significantly associated with mortality. Interestingly, among 25 patients who died during the follow-up, only 6 had been diagnosed with brain metastases. Relatively smaller number of patients with metastases to other organs compared to the group of patients with brain metastases as well as the number of patients lost to follow-up should be also taken into account; perhaps the results would be more relevant with a larger and more homogenous group of patients. However, our findings may suggest a greater impact of systemic inflammatory processes and immune dysfunction upon formation of brain metastases in comparison to their other locations.

While the systemic pro-inflammatory state is known to facilitate metastasis in general (42), organ-specific factors may influence metastatic patterns. In physiological conditions, the blood-brain barrier (BBB) limits the migration of cells and macromolecules. However, inflammation-induced disruption of the BBB - mediated by TANs and cytokines (e.g. IL-1β, TNF-α, and IL-6) - can increase vascular permeability and create a permissive environment for tumor cell infiltration into the CNS (22, 43, 44). In contrast, other organ sites may not require such inflammatory priming for metastatic colonization. This could explain the stronger correlation observed between inflammatory markers and brain metastases. Further research is needed to understand the mechanisms driving site-specific metastatic patterns and their interaction with systemic inflammation.The strengths of this study are associated with focusing on brain metastases from the lung cancer, which seem underexplored, but crucial complications of the disease. The wide and diverse range of parameters was considered in the analysis, including complete blood count and biochemical markers. The use of easily accessible and routinely measured laboratory parameters as potential prognostic markers makes this approach highly applicable to real-world settings. These findings have the potential to encourage more frequent use of these markers in clinical practice, aimed at identification of patients with greater risk of CNS involvement and undertaking individualized management strategies.

Several limitations of the study include relatively small sample size (further limited by those who were lost to follow-up) which impeded stratification by histopathological type of lung cancer. The single-center and retrospective character of the analysis may introduce selection bias and limit the generalizability of the results to more diverse populations. Also, due to the modest sample size, cross-validation methods were not applied. Therefore, the results should be interpreted with caution and considered exploratory in nature. Validation in larger, multi-center cohorts is necessary to confirm the observed associations and to establish the broader applicability of the proposed markers. An additional bias might have been caused by unrecognized brain metastases in some of the deceased patients. Due to retrospective mode of study, some data potentially relevant for their prognostic value were not available for analysis (e.g. genetic profiling of the patients, lifestyle factors including smoking). Oncogenic driver mutations, particularly EGFR and ALK, are known to increase the risk of brain metastases in non-small cell lung cancer (NSCLC) patients (45). Moreover, targeted therapies such as EGFR- and ALK-tyrosine kinase inhibitors (TKIs) are capable of crossing the blood-brain barrier and have been shown to effectively treat asymptomatic brain metastases and prolong brain-specific progression-free survival (46, 47). These factors likely influence both the incidence and timing of the metastatic burden, and their absence in our study may confound the findings. In future studies, incorporating molecular profiling and treatment stratification will be important for accurately assessing the prognostic value of systemic inflammatory markers in patients receiving targeted therapies.

## 6 Conclusions

Significant relationships were found between CBC – based indices in the patients diagnosed with lung cancer and subsequent occurrence of brain metastases. These findings may suggest the importance of inflammatory process in neoplastic activity leading to CNS involvement. CBC - based indices may be easily accessible and useful markers in identification of patients with increased risk of brain metastases, contributing to individualized diagnostic and therapeutic approach for optimal outcomes of the disease.

## Data Availability

The raw data supporting the conclusions of this article will be made available by the authors, without undue reservation.
